# The complete mitochondrial genomes and phylogenetic analysis of two Nycteribiidae bat flies (Diptera: Hippoboscoidea)

**DOI:** 10.1080/23802359.2022.2107450

**Published:** 2022-08-17

**Authors:** Megan L. Porter, Holly Lutz, Mireille Steck, Rebecca A. Chong

**Affiliations:** a School of Life Sciences, University of Hawaiʻi at Mānoa, Honolulu, HI, USA; b Department of Pediatrics, University of California San Diego, CA, USA; c Negaunee Integrative Collections Center, Field Museum of Natural History, Chicago, IL, USA

**Keywords:** Bat fly, mitogenomes, Nycteribiidae, *Dipseliopoda setosa*, *Basilia ansifera*

## Abstract

We sequenced the complete mitochondrial genomes of two bat fly species within the Nycteribiidae (Diptera: Hippoboscoidea) – *Dipseliopoda setosa* (Cyclopodiinae) and *Basilia ansifera* (Nycteribiinae). Both mitogenomes were complete and contained 13 protein-coding genes, 22 tRNAs, and two rRNAs. Relative to the inferred ancestral gene order of dipteran mitochondrial genomes, no rearrangements were identified in either species. There were large differences in size between the two genomes, with *D. setosa* having a larger genome (19,164 bp) than *B. ansifera* (16,964 bp); both species had larger genomes than two previously published Streblidae bat fly species (e.g., *Paradyschiria parvula* and *Paratrichobius longicrus*). The increased genome sizes were due to expansions in the control region and the non-coding region downstream of the light-strand origin of replication. Additional differences between the two mitogenomes included a significantly longer cox3 gene in *B. ansifera* and a longer nad1 gene in *D. setosa*. Interestingly, both genomes also had the lowest GC content (*D. setosa* – 15.9%; *B. ansifera* – 17.0%) of any available Hippoboscoidea mitochondrial genome (18.8–23.9%). These mitogenomes represent the first sequences from species within the bat fly family Nycteribiidae. The sequence data here will provide a foundation for continued studies of genome evolution more generally within obligate blood-feeding ectoparasites, and specifically for the bat flies as vectors of significant ‘bat-associated’ viruses and microorganisms.

Bat flies are obligate blood-feeding ectoparasites of bats that are taxonomically divided into two families – Streblidae and Nycteribiidae (Diptera: Hippoboscoidea). Phylogenetic studies focused on these families are sparse but suggest that while the two families form a clade, only the Nycteribiidae are monophyletic (Dittmar et al. 2006; Kutty et al. 2010). The Nycteribiidae – consisting of three subfamilies, 11 genera, and 275 species (Graciolli and Dick 2018) – are represented by predominantly Old World species. As ectoparasites of bats, the bat flies are important vectors of medically and agriculturally important ‘bat-associated’ viruses (Ramírez-Martínez et al. 2021) and microorganisms (Morse et al. 2012; Szentiványi et al. 2020), and also present an interesting evolutionary system for studying co-evolutionary patterns between hosts and ectoparasites (Porter et al. 2021). To begin building genomic resources to address outstanding questions related to bat fly systematics and evolution, here we present the first complete mitogenome sequences for two Nycteribiidae species – *Dipseliopoda setosa* Theodor, 1955 (Cyclopodiinae) and *Basilia ansifera* Theodor, 1956 (Nycteribiinae) – representing two of the three subfamilies within the family.

For each species, total genomic DNA was extracted from an entire individual using a Zymo microbiome kit; resulting DNA vouchers were deposited into the Porter Lab collections (Megan Porter, mlporter@hawaii.edu) at the University of Hawaiʻi Mānoa (accession numbers JCK10405 and JCK10356 for *D. setosa* and *B*. *ansifera,* respectively). Genomic libraries were prepared using Qiaseq FX DNA Library Kit with average insert sizes of approximately 500 bp and were sequenced using Illumina Technology at Admera Health Biopharma Services (New Jersey, USA). Adaptors were trimmed from generated reads with TrimGalore (0.6.5; Babraham Bioinformatics, 2021), and reads were assembled using NOVOplasty v4.3.1 (Dierckxsens et al. 2016). Genes from the complete mitogenomes for *D. setosa* and *B. ansifera* were annotated using the MITOS2 server (Bernt et al. 2013).

The complete mitochondrial genome of *Dipseliopoda setosa* (GenBank: MZ826151) was 19,164 bp in size with a GC content of 15.9% and contained 13 protein-coding genes (PCGs), 22 tRNAs, and two rRNA (12S and 16S) genes. The complete mitochondrial genome of *Basilia ansifera* (GenBank: MZ826150) was 16,964 bp in size with a GC content of 17.0%, and also contained 13 PCGs, 22 tRNAs, and two rRNA genes. Gene content and gene order for both genomes were consistent with the predicted ancestral insect mitochondrial genome (Cameron 2014). Nucleotide composition in both genomes was extremely AT-biased, with GC content in other Diptera mitogenomes ranging from 18.8% to 23.9%. Variation in mitochondrial genome sizes reflected significant expansions of different noncoding regions in different species: both the control region and noncoding region downstream of the origin of light-strand replication were expanded by 713 bp and 1385 bp, respectively, in *D. setosa* as compared to *B*. *ansifera*. In addition to these expansions, there were also differences in gene length between the two mitogenomes: *B. ansifera* had a larger cox3 by 21 amino acids while *D. setosa* had a longer nad1 by 27 amino acids. All other gene length differences were less than six amino acids, with the exception of nad2 and cox1 genes which are conserved in length in both species.

Mitogenomes from six species within the superfamily Hippoboscoidea were included for phylogenetic analysis, including all hippoboscoids with publicly available sequences on NCBI, as well as mitogenomes for two Glossinidae species – *Glossina austeni* (SAMN02649554) and *Glossina brevipalpis* (SAMN02647160) – that were assembled, annotated, and deposited to GenBank for this study. Representative species from Muscoidea and Oestroidea were included for evolutionary context and Ephydroidea species as outgroups. All 13 PCGs from each mitogenome were extracted and aligned using Geneious^®^ v10.2.6 with MAFFT v7.450 (Katoh and Standley 2013) to infer the phylogenetic placement of *D. setosa* and *B*. *ansifera.* We used PartitionFinder2 (Stamatakis 2006; Lanfear 2014; Lanfear et al. 2016) to identify the best partitioning scheme and nucleotide substitution models. We estimated a maximum likelihood phylogeny using RAxML (Stamatakis 2006) based on the partitioned dataset, with 1000 bootstrap replicates using the GTRGAMMAI substitution model for each partition. The phylogenetic tree suggested *D. setosa* and *B*. *ansifera* are monophyletic and that Nycteribiidae are sister to Streblidae (Figure 1).

**Figure 1.**
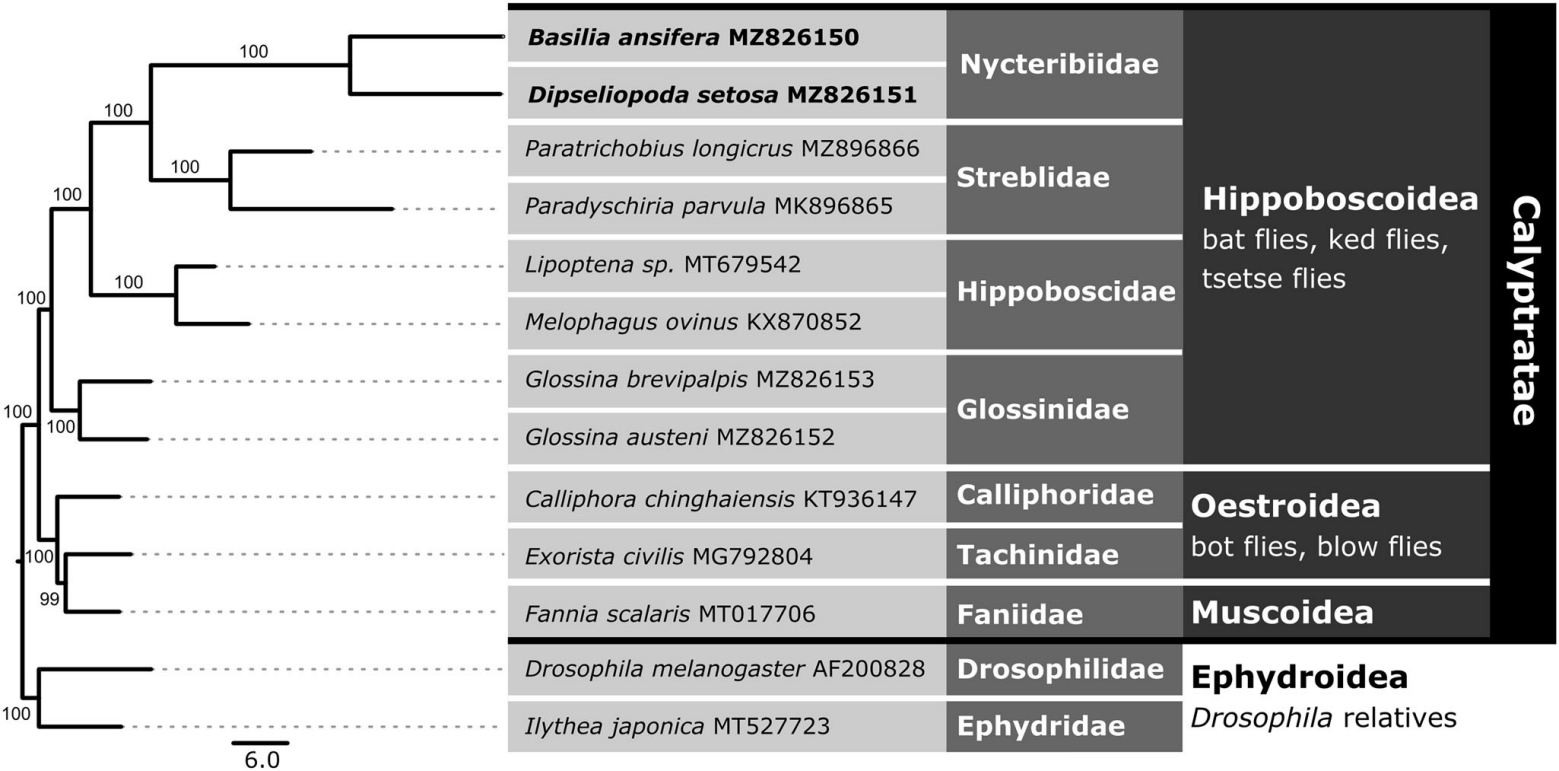
Maximum Likelihood phylogeny for *Basilia ansifera* and *Dipseliopoda setosa* based on 13 mitochondrial protein-coding genes. All nodes have a bootstrap support value ≥ 99. Genbank accession numbers follow species names, and focal species are in bold.

As the first sequenced Nycteribiidae mitogenomes, the *D. setosa* and *B*. *ansifera* mitogenomes provide a foundation for continued studies of genetic divergence among ecologically and medically important ectoparasites with distinct patterns of diversification tightly coupled with host biology. This is also the first step toward unraveling the systematics of bat flies.

## Ethics statement

Samples of *Dipseliopoda setosa* (ex. *Stenonycteris lanosus* [Chiroptera: Pteropodidae], FMNH232508) and *Basilia ansifera* (ex. *Scotoecus hindei* [Chiroptera: Vespertilionidae], FMNH232513) were collected from the Agoro-Agu Forest Reserve [3.81039, 32.92264], Uganda in 2016. Collection permits and material transfer agreements were provided by the Uganda Wildlife Authority (UWA; Ref COD/96/02), Uganda National Council for Science and Technology (UNCST; Ref NS 417).

## Authors’ contributions

HL conducted field work and collected specimens; MLP and RAC conceived and designed the study; MS generated the data; MS and RAC analyzed the data; MLP, RAC, and MS were involved in data interpretation and writing the paper; all authors revised the manuscript for intellectual content and approved the final version for publication. All authors are accountable for all aspects of the work.

## Data Availability

The genome sequence data that support the findings of this study are openly available in GenBank of NCBI at (https://www.ncbi.nlm.nih.gov/) under the accession nos. MZ826150, MZ826151, MZ826152, and MZ826153. The associated BioProject, Bio-Sample, and SRA experiment numbers are PRJNA772298, SAMN22374446 and SAMN22374447, and SRX13333010 and SRX13333011, respectively.
